# Pre-Chemoradiotherapy FDG PET/CT cannot Identify Residual Metabolically-Active Volumes within Individual Esophageal Tumors

**DOI:** 10.4172/2155-9619.1000226

**Published:** 2015-04-29

**Authors:** W Lu, S Tan, W Chen, S Kligerman, SJ Feigenberg, H Zhang, M Suntharalingam, M Kang, WD D’Souza

**Affiliations:** 1 Department of Radiation Oncology, University of Maryland School of Medicine, Baltimore, USA; 2 Department of Control Science and Engineering, Huazhong University of Science and Technology, Wuhan, China; 3 Department of Diagnostic Radiology and Nuclear Medicine, University of Maryland School of Medicine, Baltimore, USA; 4 Department of Radiation Oncology, Yeungnam University College of Medicine, Daegu, South Korea

**Keywords:** ^18^F-FDG PET/CT, Esophageal cancer, Radiation therapy

## Abstract

**Objective:**

To study whether subvolumes with a high pre-chemoradiotherapy (CRT) FDG uptake could identify residual metabolically-active volumes (MAVs) post-CRT within individual esophageal tumors. Accurate identification will allow simultaneous integrated boost to these subvolumes at higher risk to improve clinical outcomes.

**Methods:**

Twenty patients with esophageal cancer were treated with CRT plus surgery and underwent FDG PET/CT scans before and after CRT. The two scans were rigidly registered. Seven MAVs pre-CRT and four MAVs post-CRT within a tumor were defined with various SUV thresholds. The similarity and proximity between the MAVs pre-CRT and post-CRT were quantified with three metrics: fraction of post-CRT MAV included in pre-CRT MAV, volume overlap and centroid distance.

**Results:**

Eight patients had no residual MAV. Six patients had local residual MAV (SUV ≥2.5 post-CRT) within or adjoining the original MAV (SUV ≥2.5 pre-CRT). On average, less than 65% of any post-CRT MAVs was included in any pre-CRT MAVs, with a low volume overlap <45%, and large centroid distance >8.6 mm. In general, subvolumes with higher FDG-uptake pre-CRT or post-CRT had lower volume overlap and larger centroid distance. Six patients had new distant MAVs that were determined to be inflammation from radiation therapy.

**Conclusions:**

Pre-CRT PET/CT cannot reliably identify the residual MAVs within individual esophageal tumors. Simultaneous integrated boost to subvolumes with high FDG uptake pre-CRT may not be feasible.

## Introduction

Esophageal cancer remains one of the most lethal malignancies with a 5-year relative survival rate of only 17% [1]. RTOG 85-01 showed that there was a significant improvement in local control and overall survival with concurrent CRT compared with radiation therapy (RT) alone [2]. Nevertheless, local failure is still a major pattern of failure following definitive CRT, approaching 50% [3,4]. Patients with a poorer response to CRT demonstrate worse local control [5,6]. Furthermore, patients with residual tumor, assessed either clinically or pathologically after neoadjuvant CRT, have shown poorer survival [6–8]. Therefore, it is important to explore ways to improve local control.

Radiation dose escalation or boost can be one solution to improve local control and survival, as shown in the prostate cancer [9] and lung cancer [10,11]. However, when it comes to definitive CRT with current chemotherapeutic agents, the outcomes of dose escalation have been found to be no better than those of current standard dose for esophageal tumors in RTOG 94-05 [3] and for non-small cell lung cancer (NSCLC) in RTOG 06-17 [12]. Even though the reason of no benefit observed in these two trials is unclear, some investigators suggest that it may be related to the increased morbidity associated with the increased dose to organs-at-risk (OAR) in the higher-dose arm [13,14]. Therefore, dose escalation is still worth investigating by using modern intensity-modulated RT (IMRT) techniques, along with searching for novel chemotherapeutic agents. IMRT allows simultaneous integrated boost (SIB) to specific subvolumes at “higher-risk” of residual tumor within the gross tumor volume (GTV) [15]. Examples include hypoxic subvolumes or subvolumes with higher tumor cell density. Because of the smaller target volumes, the dose to these “higher-risk” subvolumes may be escalated considerably while the dose to the OAR may be kept the same as the standard techniques.

Among many molecular imaging modalities and tracers, 18F-fluorodeoxyglucose positron emission tomography (FDG-PET) is the most widely used in attempts to identify these “higher-risk” subvolumes. FDG-PET has been used for tumor staging and restaging [16], and it is the most useful modality for detecting distant metastasis [17]. The level of FDG uptake in tumor cells is a reliable marker of tumor cell glycolysis or metabolic activity and is linearly related to tumor cell proliferative activity or aggressiveness [18]. Higher uptake of FDG in pre-treatment images has been reported to be closely related to higher T- and N-stages [19–21] and poorer treatment outcomes [19,22,23]. Furthermore, presence of residual MAVs in patients after RT or CRT correlates with worse local control and survival [6,24–28]. If the locations of the residual MAVs can be identified with the high FDG uptake subvolumes on the pre-therapy PET/CT scans, SIB to these subvolumes may improve clinical outcomes [25]. In this work, we studied this question in 20 patients with esophageal cancer. To our knowledge, this is the first such study in esophageal cancer.

## Materials and Methods

### Patients

This retrospective study was approved by our institutional review board. The cohort included 20 consecutive patients (median age, 64 years) with esophageal cancer, who underwent trimodality therapy (CRT plus surgery) from 2006 to 2009 and had PET/CT scans both before and after CRT (Table 1). Staging was according to AJCC Cancer Staging Manual sixth edition [29], where M1a is extensive local–regional lymph node disease without distant metastasis.

### PET/CT imaging

Pre-CRT PET/CT imaging was performed 32 days (median, range 19–88 days) before CRT, and post-CRT imaging was performed 33 days (median, range 28–48 days) after completion of CRT but before surgery. All PET/CT studies were performed with an integrated 16-slice Gemini PET/CT scanner (Philips Medical Systems; Cleveland, OH). Following an institutional standard protocol, each patient fasted for a minimum of 4 h before intravenous injection of 12–14 mCi ^18^F-FDG.

Whole-body PET and CT imaging was started 60 min (median 60 min, range 50–70 min) after tracer injection. The patient’s arms were abducted during the scan. Patient breathed quietly and respiratory gating was not applied. PET images were attenuation corrected and reconstructed with a maximum likelihood algorithm. Resolution for PET images was 4.0×4.0×4.0 mm^3^ and for CT images was 0.98×0.98×4.0 mm^3^.

### Chemoradiotherapy

All patients were treated with external-beam RT with concurrent chemotherapy. A radiation dose of 50.4 Gy (1.8 Gy/day, 5 days/week) was delivered using CT simulation and 3D or IMRT treatment planning. The GTV was manually delineated by including all known disease seen on PET-CT scan and EUS/EGD. The margins from GTV to the clinical target volume (CTV) were 4 cm superiorly and inferiorly, and 1 cm axially. The margin from CTV to the planning target volume (PTV) was 1 cm uniformly.

Chemotherapy consisted of cisplatin (100 mg/m^2^) administered intravenously on day 1 of week 1 and 5 and 5-fluorouracil (1,000 mg/m^2^) administered daily as a continuous intravenous infusion over 4 consecutive days in week 1 and 5.

### Pathologic assessment

Surgical resection was performed 25 days (median, range 6–126 days) after the post-CRT PET/CT, and 59 days (median, range 39–159 days) after CRT. The resected surgical specimen was submitted to the same pathologist (blinded to the study hypothesis) for evaluation.

The specimen was microscopically examined, and semi-quantitatively categorized into 1 of 3 groups: pathologic complete response (pCR), microscopic residual disease (mRD), or gross residual disease (gRD), according to the amount of residual viable carcinoma observed in relation to volumes of fibrosis [30].

### Image analysis

A rigid 3D registration technique (VersorRigid3DTransform in ITK) [31] was used to register the post-CRT CT to the pre-CRT CT by maximizing their normalized correlation. The rotation and translation parameters are represented by a vesor and a vector, respectively.

To achieve higher registration accuracy in the tumor region, registration was conducted within a rectangular chest region excluding patient’s arms and head. Next, the results were visually examined and adjusted if deemed necessary by a radiologist (SK).

The resulting registration transform was directly applied to register the post-CRT PET to the pre-CRT PET. The registration algorithm was optimized and tested on simulated CT images with known rotations up to 10° and/or translations up to 10 cm. The registration error was <0.5 voxel in the simulation study. In patients, no obvious misalignments were observed.

Following image registration, the original and residual MAVs were delineated using a region-growing method [32] with a threshold of SUV ≥2.5 on the pre-CRT and post-CRT PET images, respectively. This threshold has been widely used for classifying FDG uptake in various cancers [33] and has been shown to delineate esophageal tumors with reasonable accuracy [34–36].

Similarly, a total of seven MAVs pre-CRT and four MAVs post-CRT with high FDG uptakes were defined using thresholds of SUV 2.5 (original MAV), SUV 5.0, 34%, 40%, 50%, 60%, and 70% of the maximal SUV (SUV_max_) pre-CRT, and SUV 2.5 (residual MAV), 70%, 80% and 90% of SUV_max_ post-CRT, respectively.

To quantify the similarity and proximity between each of the seven MAVs pre-CRT and the four MAVs post-CRT, the following metrics were computed.

**Figure 1:**
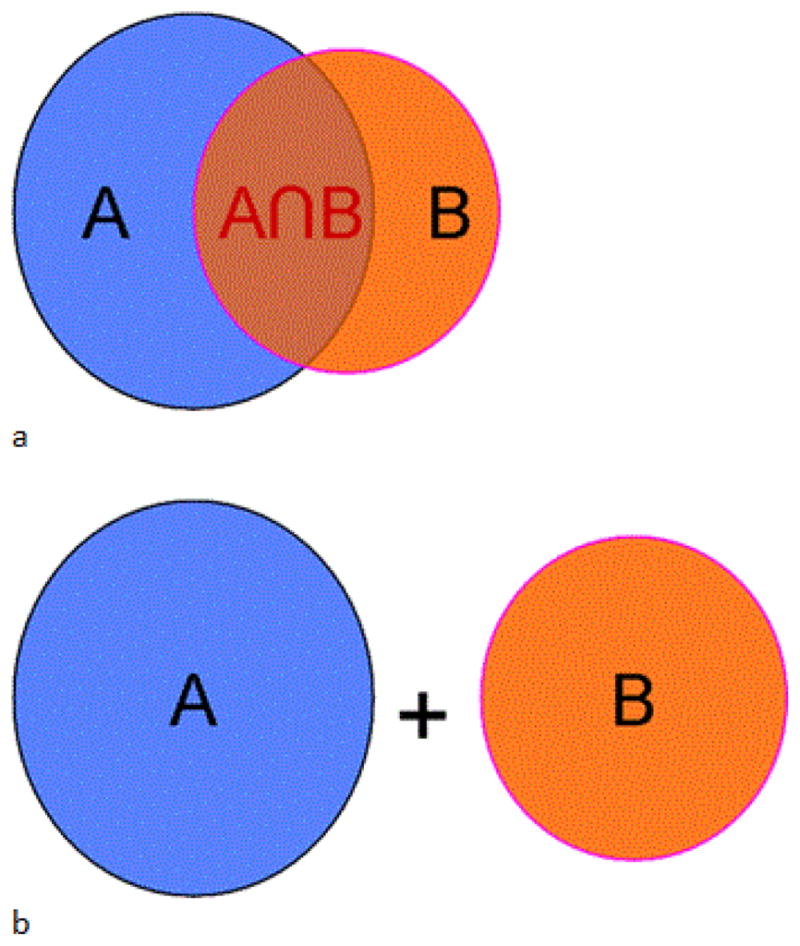
Illustration of the two overlap metrics. A B is the interception of A and B (a), and A+B is the sum of A and B (b). $\mathrm{BinA}=\frac{A\cap B}{B}$, and $\mathrm{Overlap}=2\cdot \frac{A\cap B}{A+B}$

1. Fraction of B Included in A (BinA, Figure 1):
  $$\mathrm{BinA}=\frac{A\cap B}{B}$$
2. Volume Overlap quantified by Dice Coefficient, (Overlap, Figure 1):
  $$\mathrm{Overlap}=2\cdot \frac{A\cap B}{A+B}$$
3. Centroid Distance between A and B,
  where A denotes an MAV in pre-CRT PET/CT and B denotes an MAV in registered post-CRT PET/CT.

Both Volume Overlap and *BinA* fall in the range of [0,1], and the larger the value, the higher is the similarity and proximity of A and B. In contrast, the larger the Centroid Distance, the lower is the proximity.

## Results

### Tumor FDG uptake and MAVs

All 20 primary tumors showed above-background metabolic activity (SUV_max_ ≥ 2.5) on the pre-CRT PET/CT scans, with a mean SUV_max_ of 8.7 (median 8.3, range 3.0–19.0), and mean original MAVs of 44.7 cm^3^ (median 47.0, range 4.8–100.4). All tumors showed heterogeneous FDG uptake pattern where the uptake varies in space and the highest-uptake subvolume was not necessarily in the center of the tumor. The volume of the original MAVs was not significantly associated with pre-CRT SUV_max_, although their Spearman correlation coefficient was r=0.41, with p=0.08.

### Comparison between patients with residual MAVs and patients without

Among the 20 patients, six had local (within or adjoining the original MAVs, Volume Overlap >0) residual MAVs on the post-CRT PET/CT scans. Another six patients had new MAVs post-CRT (2 in liver and 4 in other parts of esophagus) that were distant (Volume Overlap=0) from the original MAVs pre-CRT. The cause of all new distant MAVs was determined to be inflammation from RT based on further follow-up imaging and pathology studies, by a Nuclear Medicine physician (WC). Five patients demonstrated no MAVs (post-CRT SUV_max_<2.5). Three patients showed minimal residual MAVs with post-CRT SUV_max_ values of 2.5, 3.0, 2.5, and volumes of 0.1, 0.4, and 1.4 cm^3^, respectively. Because of the small volumes and the mildly above-background FDG uptake, they were considered not to have residual MAVs.

There were no statistically significant difference in the volume of the original MAVs (47.1 vs. 43.7 cm^3^), or pre-CRT SUV_max_ (10.7 vs. 7.8), or decrease in SUV_max_ (4.5 vs. 6.4) between patients (n=6) with local residual MAVs and patients (n=14) without local residual MAVs (Wilcoxon test, p=0.84, 0.18, and 0.49, respectively). Similarly, there were no statistically significant difference in the volume of the original MAVs (38.1 vs. 54.7 cm^3^), or pre-CRT SUV_max_ (9.0 vs. 8.2), or decrease in SUV_max_ (5.1 vs. 6.9) between patients (n=12) with local or distant residual MAVs and patients (n=8) without any residual MAVs (Wilcoxon test, p=0.34, 0.52, and 0.34, respectively).

### Similarity and proximity between MAVs pre-CRT and MAVs post-CRT

**Figure 2:**
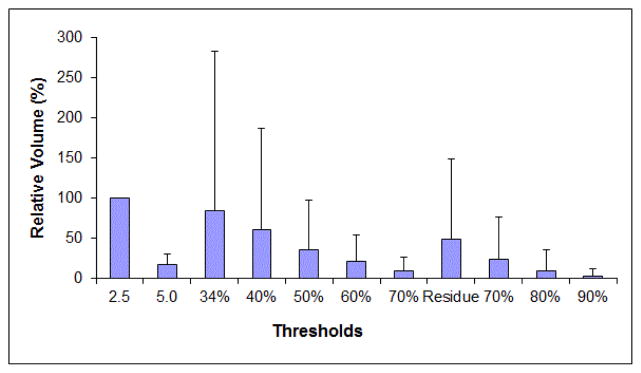
Relative volumes of the seven MAVs pre-CRT (SUV thresholds 2.5, 5.0, 34%, 40%, 50%, 60%, and 70% of SUV_max_) and the four MAVs post-CRT (SUV thresholds 2.5, 70%, 80%, and 90% of SUV_max_). Normalized to the volume of the original MAV. Error bar represents one standard error.

The following analyses are only for the six patients who had local residual MAVs. Figure 2 shows the relative volumes of the seven MAVs pre-CRT and the four MAVs post-CRT. Table 2 gives BinA, Volume Overlap, and Centroid Distance between the original MAVs and the residual MAVs. From pre-CRT to post-CRT, the tumor mean SUVmax decreased from 10.7 to 6.1 (43% decrease), and the mean MAV reduced from 47.1 cm^3^ (original MAV) to 23.0 cm^3^ (residual MAV, 51% reduction). Large variations were observed among the patients. On average, only 60% of the residual MAVs were included in the original MAVs while the overlap between the two was even lower at 37%. The centroid distance between them was 11.9 mm.

**Figure 3:**
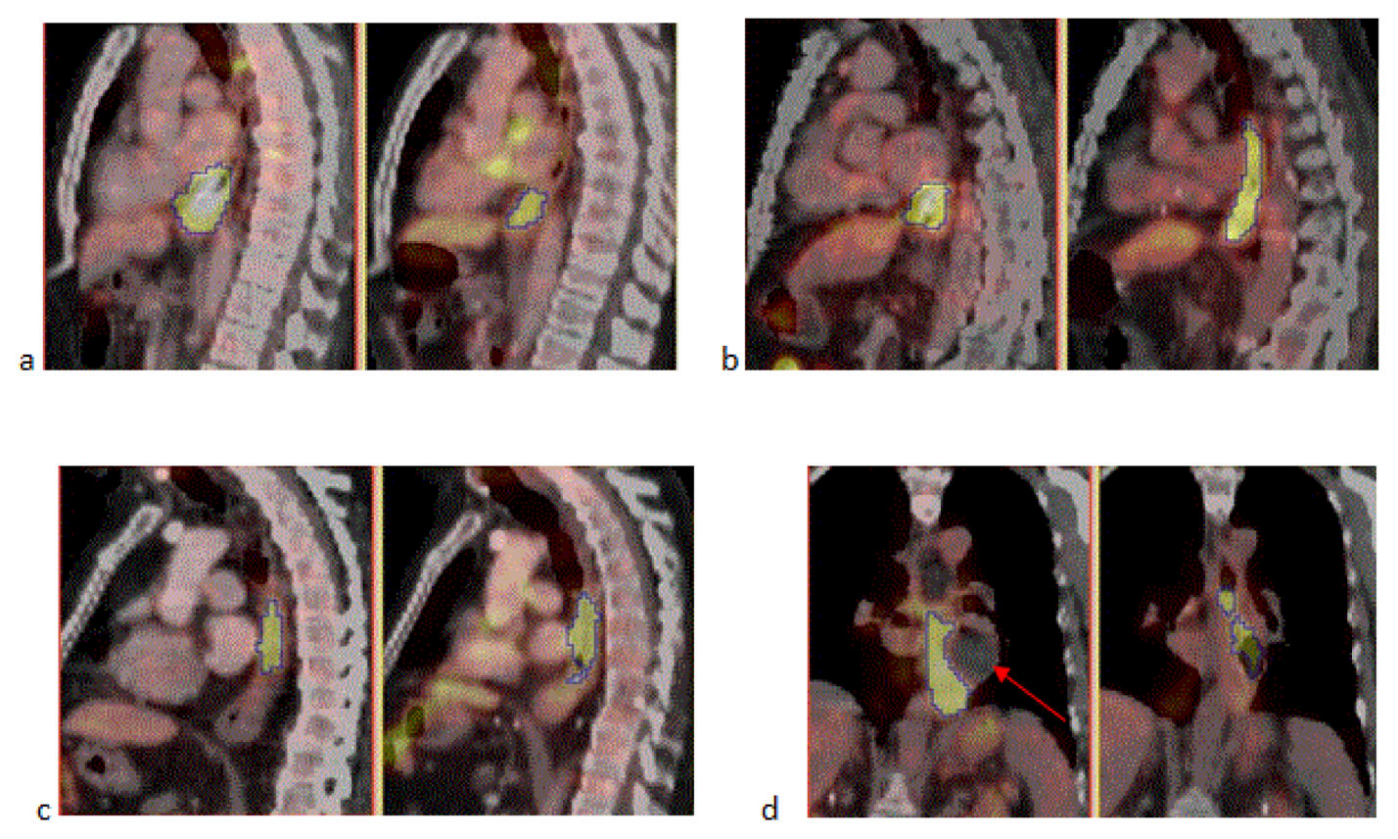
FDG PET/CT images of four patients pre-CRT (left) and post-CRT (right). The contours indicate the original MAVs on pre-CRT images and residual MAVs on post-CRT images, respectively. (a) Patient 6, BinA=100%, overlap=33%, (b) Patient 4, BinA=43%, overlap=44%, (c) Patient 3, BinA=37%, overlap=53%, (d) Patient 2, BinA=14%, overlap=5%.

Figure 3 shows representative images of four types of spatial relationships between the original MAVs and residual MAVs. Figure 3a is an example of three patients (Patients 1,5, and 6) who showed local residual MAVs with decreased SUV. They had moderate to high BinA (91%, 74% and 100%) but the overlaps were low (62%, 22% and 33%). Figure 3b showed residual MAVs with decreased SUV that extended into the neighborhood of the original MAVs. It had low BinA (43%) and overlap (44%). Figure 3c showed residual MAVs with unchanged SUV that extended beyond the original MAVs. It had a low BinA (37%) and overlap (53%). Figure 3d showed residual MAVs with decreased SUV. Though the residual MAVs resided at similar levels in the esophagus as the original MAVs, it had the lowest BinA of 14% and overlap of 5%. On pre-CRT images the tumor infiltration along the wall of the esophagus was eccentric leading to focal dilation of the esophageal lumen (arrow) in the opposite direction, while on post-CRT image this dilated lumen was almost completely resolved. Because of this large change in non-FDG-avid tissue near the tumor, both BinA and overlap were very low.

**Figure 4:**
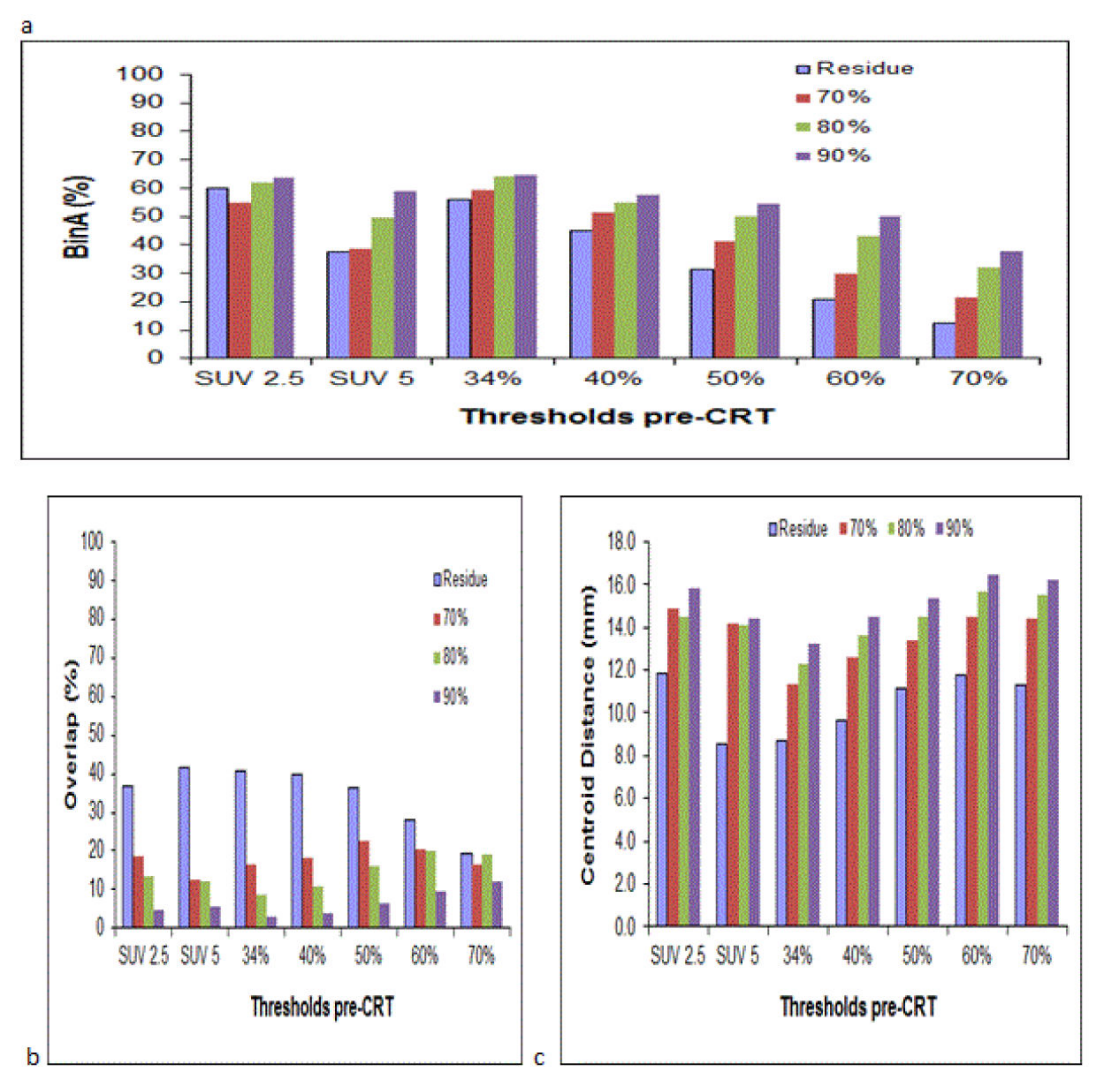
The average BinA (a), Volume Overlap (b), and centroid distance (c) between the seven MAVs pre-CRT and the four MAVs post-CRT.

As depicted in Figure 4, the average BinAs between the seven MAVs pre-CRT and the four MAVs post-CRT were all less than 65%, and the average overlap all less than 45%, while the average centroid distances were all larger than 8.6 mm. The 70%, 80% and 90% SUV_max_ MAVs post-CRT were typically enclosed completely by the residual MAV (defined with a threshold of SUV 2.5). Therefore, the average BinAs for the 70%, 80% and 90% SUV_max_ MAVs post-CRT were typically higher than those for the residual MAV. On the contrary, the average overlap for the 70%, 80% and 90% SUV_max_ MAVs post-CRT were lower (all less than 23%), while the average centroid distances were larger (all larger than 11.4 mm) than those for the residual MAV. In general, subvolumes with higher FDG-uptake pre-CRT or post-CRT had lower volume overlap and larger centroid distance. These results suggested that none of the seven MAVs pre-CRT corresponded well with or could identify any of the four MAVs post-CRT.

## Discussion

None of the parameters: volume of the original MAV, pre-CRT SUV_max_, or decrease in SUV_max_ was significantly different between patients with and without residual MAV post-CRT. Therefore it is impossible to predict which patients will have a residual MAV.

Of all 20 patients, six had local residual MAVs. Among the six patients, only three had moderate to high BinA (fraction of post-CRT MAV included in the pre-CRT MAV). Even for these three patients, the overlaps between the pre- and post-CRT MAVs were low and the centroid distances were large. These results suggest that the high FDG uptake subvolumes on the pre-CRT PET/CT can not reliably identify the MAVs post-CRT. Because the size and location of the high FDG uptake subvolumes changed considerably during the treatment, SIB to these subvolumes on the pre-CRT PET/CT may not be feasible for patients with esophageal cancer. Instead adaptive dose escalation strategy, i.e. re-imaging and re-planning boost dose to FDG-avid subvolumes during treatment, is warranted though it has not been established.

Aerts et al. performed a similar study in NSCLC treated with CRT or RT alone [25]. They found that 22 of 55 patients had residual FDG uptake post-therapy that highly corresponded (Overlap Fraction >91%; see below) with GTV pre-therapy. They concluded that pre-therapy FDG PET/CT allows for identification of residual MAVs in NSCLC. Our results and conclusion in esophageal cancer were not comparable. There are several possible explanations for the low correlations between high FDG uptake subvolumes pre-CRT and post-CRT in esophageal cancer. Firstly, up to some weeks after 50.4 Gy of RT, a physiologic FDG uptake, which can be quite intense, may be seen in the irradiated esophagus due to inflammation. In the example patients given in Figure 3b and 3c (both had gRD), this might be an explanation for the low BinA and overlap. In general, it is difficult to tell whether the local residual FDG uptake seen on the post-CRT PET/CT is due to inflammation or persistent malignancy or both. In Aerts’ study, only one of the 28 NSCLC patients showed FDG avid inflammation and was excluded from that study. Secondly, in esophageal cancer, large change in non-FDG-avid tissue near the tumor, such as the esophageal lumen shown in Figure 3d, could lead to very low BinA and overlap between the MAVs pre- and post-CRT. In NSCLC, this impact was considered to be much smaller. Thirdly, Aerts et al. excluded six patients (21%) from the 28 patients who had residual FDG uptake because of progressive disease (one patient), large tumor deformation (two patients) or difficulty in tumor delineation (three patients). These patients might have a lower overlap. Lastly, Aerts et al. evaluated the overlap between two volumes with

$$\mathrm{Overlap}\;\mathrm{Fraction}\;(OF)=\frac{A\cap B}{\min(A,B)}=\max(\frac{A\cap B}{A},\frac{A\cap B}{B})=\max(\mathrm{AinB},\mathrm{BinA}).$$

OF is always greater than or equal to BinA and Volume Overlap (by Dice Coefficient), as used in our work (Table 2). If either A is much smaller than B (for example Patient 3) or B is much smaller than A (Patient 6), OF tends to overestimate their overlap. If B is much smaller than A (Patient 6), BinA also tends to overestimate their overlap. In both cases, the Volume Overlap provides a more accurate quantification of the overlap by dividing the interception by the sum of A and B, thus removing the bias when using A or B alone as base [37]. Aerts et al. had to exclude one patient with progressive disease because the residual MAV enclosed the original MAV completely, resulting in an unreliable OF (of 1.0).

In this case, Volume Overlap can still provide a reliable quantification of the overlap (<1.0). We suggest that Volume Overlap and BinA should be used instead of OF for such studies. In our study, 17 of the 20 patients were adenocarcinoma and only 3 were squamous cell carcinoma (SCC). One of the 3 patients with SCC had local residual MAVs on the post-CRT PET/CT scans (Patient 2 in Figure 3d and Table 2). This patient had the lowest BinA and overlap due to large change in nearby non-FDG-avid tissue. Koshy et al. found that patients with SCC were more likely to achieve a pCR to neoadjuvant therapy when compared with patients with adenocarcinoma. Therefore, it would be interesting to study our question separately for these two histology types in a larger dataset.

There are a few limitations of this study. Firstly, the surrogate endpoint, presence of residual MAVs in the tumor, is not equivalent to presence of residual tumor, nor does a metabolic complete response equal a cure. However, as shown in the literature [25–28], pathologic response and/or survival of patients with residual MAVs in the tumor is significantly worse than those without. These results support the clinical validity of the surrogate endpoint. Secondly, errors in image registration between the pre- and post-CRT scans play a part in the measured overlap and centroid distance between the two volumes. We used a rigid registration algorithm in ITK, and validated in simulation study that the registration error was less than half voxel (0.5 mm in transverse plane and 2 mm in superior-inferior direction). In patients, we visually verified the registration and did not observe obvious misalignments. We therefore consider the effect of registration errors as small, compared to the measured large centroid distance (>8.6 mm). Deformable registration algorithms may compensate therapy-induced deformations in tumor and surrounding tissues so that the overlap could be higher. However, these deformations can not be accounted for by the pre-treatment SIB strategy. Instead, adaptive planning strategy is warranted. Therefore deformable registration algorithms were not used in Aerts et al. [25] or this study. Another limitation is that this is a study of a small patient cohort, we are in the process of extending this study to a large patient cohort collected by another institution.

Future works on the use of PET/CT for radiation dose escalation in esophageal cancer will likely require re-imaging, response evaluation, and re-planning during the course of treatment (i.e. adaptive radiotherapy planning), similar to RTOG1106 trial in NSCLC (http://www.rtog.org/ClinicalTrials/ProtocolTable/StudyDetails.aspx?study=1106). The usefulness of PET-response-guided treatment strategy for esophageal cancer has been demonstrated by the MUNICON phase II trial in chemotherapy [38].

## Conclusion

The results of this study suggest that pre-CRT PET/CT cannot reliably identify the residual metabolically-active volumes in esophageal cancer. Simultaneous integrated boost to subvolumes with high FDG uptake on the pre-CRT PET/CT may not be feasible in patients with esophageal cancer.

## Figures and Tables

**Table 1:** Patient characteristics (n=20).

Characteristic	No. of patients
**Sex**	
Male	18
Female	2
Primary site	
Proximal	0
Distal	20
Mid	0
Throughout	0
**Histology**	
Squamous cell carcinoma	3
Adenocarcinoma	17
Histologic grade	
Well differentiated	3
Moderately differentiated	10
Poorly differentiated	5
Unknown	2
**Clinical stage**	
T1	0
T2	2
T3	18
T4	0
N0	6
N1	14
M0	18
M1a	2
**Pathologic stage**	
ypT0	5
ypT2	8
ypT3	6
ypTx	1
ypN0	14
ypN1	6
**Pathologic response**	
Pathologic complete response	5
Microscopic residual disease	4
Gross residual disease	11

**Table 2:** Similarity and proximity between the original MAV and residual MAV for the six patients with local residual MAV.

Patie nt	Stage	Pathologic Tumor Response	SUVmax Pre-CRT	SUVmax Post-CRT	Original MAV (cm^3^)	Residual MAV (cm^3^)	Bin A	Volume Overlap by Dice	Overlap Fraction	Centroid Distance (mm)
**1**	T3N1 M0	gRD	19	14	55.4	28.6	0.91	0.62	0.91	10.1
**2**	T3N0 M0	mRD	6.1	4	63.1	12.5	0.14	0.05	0.14	23
**3**	T2N0 M0	gRD	4.5	5	10.6	29.1	0.37	0.53	1	2.9
**4**	T3N1 M0	gRD	11.7	6.4	43.3	46.8	0.43	0.44	0.46	18.6
**5**	T3N1 M0	pCR	9.4	3.4	45.8	7.7	0.74	0.22	0.74	9.8
**6**	T3N1 M0	pCR	13.2	3.9	64.3	13	1	0.33	1	6.7
**Mean**			10.7	6.1	47.1	23	0.6	0.37	0.71	11.9
